# Crystal structure, Hirshfeld surface analysis and computational study of the 1:2 co-crystal formed between *N*,*N*′-bis­[(pyridin-4-yl)meth­yl]ethanedi­amide and 3-chloro­benzoic acid

**DOI:** 10.1107/S2056989020006568

**Published:** 2020-05-19

**Authors:** Sang Loon Tan, Edward R. T. Tiekink

**Affiliations:** a Research Centre for Crystalline Materials, School of Science and Technology, Sunway University, 47500 Bandar Sunway, Selangor Darul Ehsan, Malaysia

**Keywords:** crystal structure, oxalamide, benzoic acid derivative, hydrogen bonding, Hirshfeld surface analysis, computational chemistry

## Abstract

The asymmetric unit of the title 1:2 co-crystal, C_14_H_14_N_4_O_2_·2C_7_H_5_ClO_2_, comprises a half-mol­ecule of oxalamide (^4^ *L*H_2_), being located about a centre of inversion, and a mol­ecule of3-chloro­benzoic acid (3-ClBA) in a general position. From symmetry, the ^4^ *L*H_2_ mol­ecule has a (+)anti­periplanar conformation with the 4-pyridyl residues lying to either side of the central, planar C_2_N_2_O_2_ chromophore with the dihedral angle between the core and pyridyl ring being 74.69 (11)°; intra­molecular amide-N—H⋯O(amide) hydrogen bonds are noted. The 3-ClBA mol­ecule exhibits a small twist as seen in the C_6_/CO_2_ dihedral angle of 8.731 (12)°. In the mol­ecular packing, three-mol­ecule aggregates are formed *via* carb­oxy­lic acid-O—H⋯N(pyrid­yl) hydrogen bonding. These are connected into a supra­molecular tape along [111] through amide-N—H⋯O(carbon­yl) hydrogen bonding. Additional points of contact between mol­ecules include pyridyl and benzoic acid-C—H⋯O(amide), methyl­ene-C—H⋯O(carbon­yl) and C—Cl⋯π(pyrid­yl) inter­actions so a three-dimensional architecture results. The contributions to the calculated Hirshfeld surface are dominated by H⋯H (28.5%), H⋯O/O⋯H (23.2%), H⋯C/C⋯H (23.3%), H⋯Cl/Cl⋯H (10.0%) and C⋯Cl/C⋯Cl (6.2%) contacts. Computational chemistry confirms the C—Cl⋯π inter­action is weak, and the importance of both electrostatic and dispersion terms in sustaining the mol­ecular packing despite the strong electrostatic term provided by the carb­oxy­lic acid-O—H⋯N(pyrid­yl) hydrogen bonds.

## Abstract 2

In the title 1:2 co-crystal a three-mol­ecule aggregate, *i.e*. ^4^ *L*H_2_.2(3-ClBA), is formed *via* carb­oxy­lic acid-O—H⋯N(pyrid­yl) hydrogen bonding. The three-mol­ecule aggregates are connected into a supra­molecular tape along [111] by amide-N—H⋯O(carbon­yl) hydrogen bonding.

## Chemical context   

Herein, the X-ray crystal structure determination of the 1:2 co-crystal formed between bis­(pyridin-4*-*ylmeth­yl)ethanedi­amide and 3-chloro­benzoic acid, (I), is described. The present crystallographic study continues recent studies into the structural chemistry of the isomeric bis­(pyridin-*n-*ylmeth­yl)ethanedi­amide mol­ecules, *i.e*. species with the general formula *n*-NC_5_H_4_CH_2_N(H)C(=O)C(=O)CH_2_C_5_H_4_N-*n*, for *n* = 2, 3 and 4, and hereafter, abbreviated as *^n^L*H_2_ (Tiekink, 2017 ▸). These mol­ecules have inter­est as co-crystal co-formers as they possess both hydrogen-bonding donating and accepting sites, *i.e*. amide and pyridyl functionalities. A particular focus of these studies has been upon co-crystals formed with carb­oxy­lic acids (Arman *et al.*, 2012 ▸, 2014 ▸; Tan, Halcovitch *et al.*, 2019 ▸; Tan & Tiekink, 2019 ▸), directed by the reliability of the carb­oxy­lic acid-O—H⋯N(pyrid­yl) synthon (Shattock *et al.*, 2008 ▸). A common thread of recent investigations has been upon benzoic acid (Tan & Tiekink, 2020*a* ▸) and derivatives (Syed *et al.*, 2016 ▸), in particular halide-substituted species (Tan & Tiekink, 2020*b* ▸) in order to probe for the possibility of competing/complementary halogen-bonding inter­actions. In connection with this theme, this report describes the crystal and mol­ecular structures of (I), along with a detailed analysis of the supra­molecular association through the calculation of the Hirshfeld surface and computational chemistry.
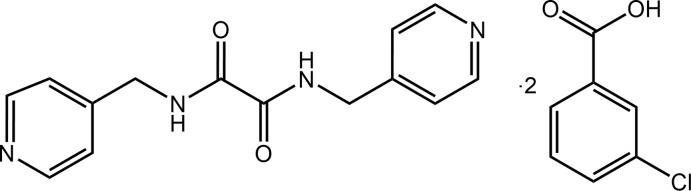


## Structural commentary   

**Figure 1:**
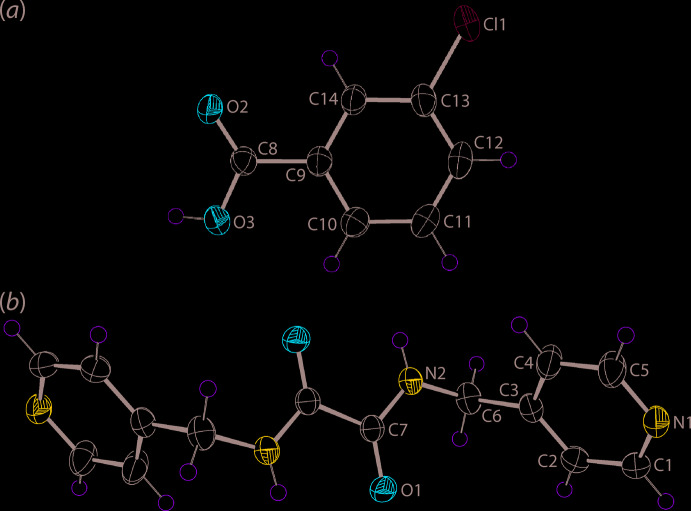
The mol­ecular structures of the constituents of co-crystal (I) showing the atom-labelling scheme and displacement ellipsoids at the 50% probability level: (*a*) the 3-chloro­benzoic acid mol­ecule and (*b*) the centrosymmetric *N*,*N*′-bis­[(pyridin-4-yl)meth­yl]oxalamide mol­ecule with the unlabelled atoms related by the symmetry operation (i) 1 − *x*, − *y*, − *z*.

The asymmetric unit of (I) comprises a mol­ecule of 4-chloro­benzoic acid (3-ClBA) in a general position and one-half mol­ecule of ^4^ *L*H_2_, being disposed about a centre of inversion, Fig. 1 ▸. In the acid, 3-ClBA, there is a definitive disparity in the C8—O2 [1.225 (2) Å] and C8—O3 [1.308 (2) Å] bond lengths entirely consistent with the localization of the acidic proton on the O3 atom. This is also borne out in the angles subtended at the C8 atom with the widest angle involving the oxygen atoms [O2—C8—O3 = 123.38 (17)°] and the narrowest involving the atoms connected by a single bond [O3—C8—C9 = 114.23 (15)°]. A small twist in the mol­ecule is evident as seen in the dihedral angle of 8.731 (12)° formed between the CO_2_/C_6_ residues; the O2—C8—C9—C10 torsion angle = 171.79 (19) Å.

The ^4^ *L*H_2_ mol­ecule is situated about a centre of inversion so the central C_2_N_2_O_2_ chromophore is constrained to be planar. As is normal for *^n^L*H_2_ mol­ecules (Tiekink, 2017 ▸), the central C7—C7^i^ [1.539 (3) Å; symmetry code: (i) 1 − *x*, − *y*, − *z*] bond length is considered long, an observation ascribed to the electronegative substituents bound to the *sp* ^2^-C7 atom. The conformation of the ^4^ *L*H_2_ mol­ecule is (+)anti­periplanar so the 4-pyridyl residues lie to either side of the planar region of the mol­ecule. The dihedral angle between the central core and the N1-pyridyl ring is 74.69 (11)°. Owing to the *anti*-disposition of the amide groups intra­molecular amide-N—H⋯O(amide) hydrogen bonds are formed which complete *S*(5) loops, Table 1 ▸.

## Supra­molecular features   

**Figure 2:**
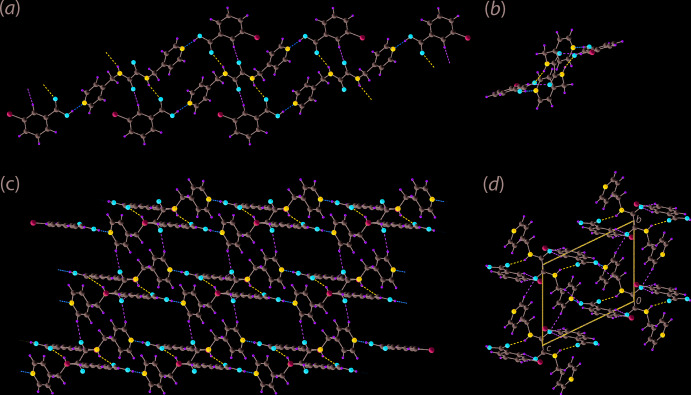
Mol­ecular packing in the crystal of (I): (*a*) a view of the supra­molecular tape comprising three-mol­ecule aggregates (sustained by carb­oxy­lic acid-O—H⋯N(pyrid­yl) hydrogen bonding shown as orange dashed lines) linked by amide-N—H⋯O(carbon­yl) hydrogen bonding (blue dashed lines) and supporting benzoic acid-C—H⋯O(carbon­yl) inter­actions (green dashed lines), (*b*) an end-on view of the tape viewed down [111], (*c*) a view of the supra­molecular layer whereby the tapes of (*a*) are linked by short pyridyl-C—H⋯O(carbon­yl) inter­actions and (*d*) a view of the unit-cell contents down the *a* axis.

The most distinctive feature of the mol­ecular packing is the association between ^4^ *L*H_2_ and two symmetry-related 3-ClBA mol­ecules *via* carb­oxy­lic acid-O—H⋯N(pyrid­yl) hydrogen bonding, Table 1 ▸, to generate a three-mol­ecule aggregate. These three-mol­ecule aggregates are connected into a linear tape along [111] *via* amide-N—H⋯O(carbon­yl) hydrogen bonds Fig. 2 ▸(*a*). These give rise to 22-membered {⋯NC_4_NH⋯OCOH}_2_ synthons. Additional stability to the hydrogen-bonding arrangement is provided by supporting benzoic acid-C14—H⋯O(amide) inter­action which lead to non-symmetric 10-membered {⋯HC_3_O⋯HNC_2_O}_2_ synthons, which flank the larger 22-membered rings. Further, a complementary C—Cl⋯π(pyrid­yl) contact is noted, as detailed in Table 1 ▸. A survey of the literature (Imai *et al.*, 2008 ▸) as well as the Cambridge Structural Database (Groom *et al.*, 2016 ▸) shows that the average Cl⋯π distance is about 3.6 Å, which is shorter than the contact distance in (I). An end-on view of the tape is shown in Fig. 2 ▸(*b*). The tapes are connected into a supra­molecular layer by relatively short pyridyl-C1—H⋯O(amide) contacts, Fig. 2 ▸(*c*). A three-dimensional architecture results when benzoic acid-C12—H⋯O(amide) and methyl­ene-C—H⋯O(carbon­yl) inter­actions are taken into consideration, Fig. 2 ▸(*d*). In this scheme, the amide-O1 atom participates in three pivotal C—H⋯O inter­actions.

## Hirshfeld surface analysis   

**Figure 3:**
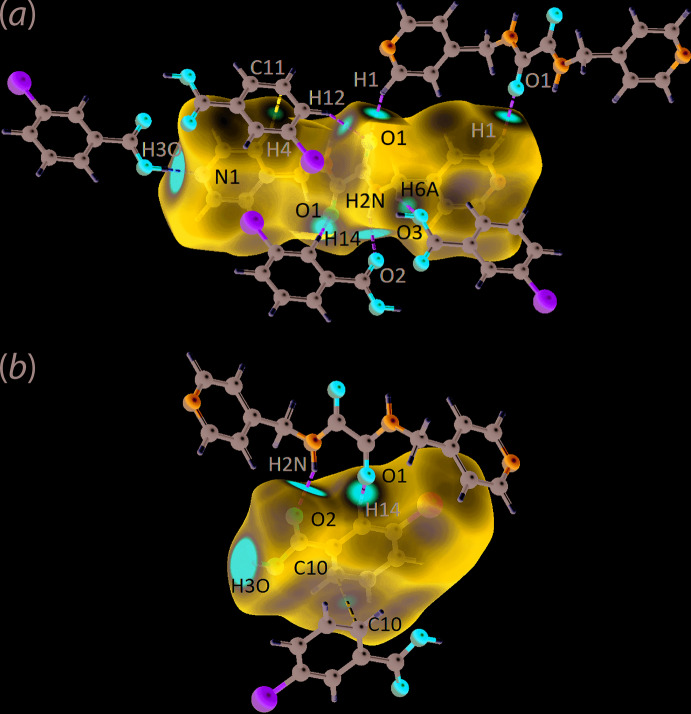
The *d*_norm_ maps plotted within the range of −0.2015 to 1.0590 arbitrary units for (*a*) ^4^ *L*H_2_ and (*b*) 3-ClBA, showing O—H⋯N (yellow dashed lines), (N,C)—H⋯O (green dashed lines), C—H⋯C (blue dashed lines) and C⋯C (light-purple dashed lines) close contacts as indicated by the corresponding red spots of varying intensity.

The Hirshfeld surface analysis was performed for the three-mol­ecule aggregate of (I), *i.e*. that sustained by the carb­oxy­lic acid-O—H⋯N(pyrid­yl) hydrogen bonds, and for the individual components, *viz*. the full mol­ecule of ^4^ *L*H_2_ and 3-ClBA, with the use of *CrystalExplorer17* (Turner *et al.*, 2017 ▸) and based on established methods (Tan, Jotani *et al.*, 2019 ▸). As shown in the images of Fig. 3 ▸, the analysis reveals there are several red spots of variable intensity observed on the *d*_norm_ maps calculated for ^4^ *L*H_2_ and 3-ClBA. These are indicative of close contact distances shorter than the van der Waals radii (Spackman & Jayatilaka, 2009 ▸). Specifically, red spots with intensity in decreasing order are observed for hydroxyl-O3—H3*O*⋯N1(pyrid­yl), amide-N2—H2*N*⋯O2(carbon­yl), pyr­id­yl-C1—H1⋯O1(amide), benzene-C14—H14⋯O1(amide), benzene-C12—H12⋯O1(amide) and methyl­ene-C6—H6*A*⋯O3(hydrox­yl); the *d*_norm_ distances for these short contacts are given in Table 2 ▸. While the identified close contacts are consistent with those obtained from *PLATON* analysis (Spek, 2020 ▸), additional red spots are noted for pyridyl-C4—H4⋯C11(benzene) as well as benzyl-C10⋯C10(benzene), albeit with relatively weaker intensity than the other inter­actions mentioned above. As for the C13–Cl1⋯π(N1,C1–C5) contact, Table 2 ▸, the Hirshfeld surface analysis reveals only a faint-blue spot around the tip of Cl1 in Fig. 3 ▸(*b*) indicating the contact distance that is slightly less than the sum of the van der Waals radii (Spackman & Jayatilaka, 2009 ▸).

**Figure 4:**
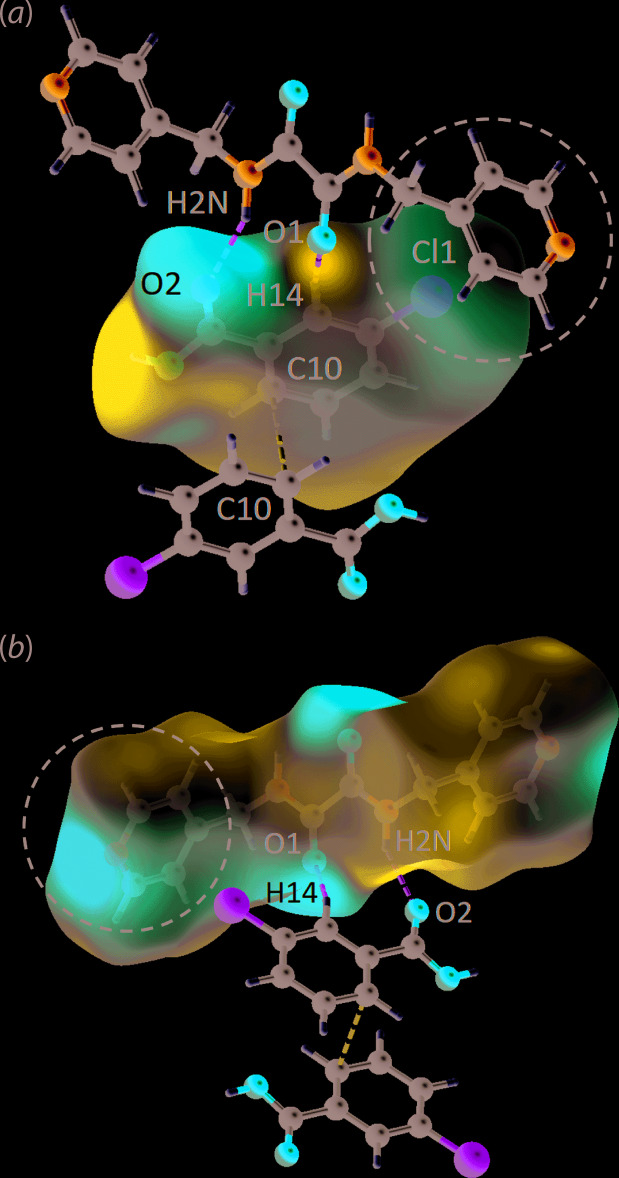
The electrostatic potential mapped onto the Hirshfeld surfaces within the isosurface value of −0.0481 to 0.0854 atomic units for (*a*) 3-ClBA and (*b*) ^4^ *L*H_2_. The circles highlight the inter­action between the Cl1 atom, through the σ-hole region, and π-hole of the pyridyl ring.

To verify the nature of the Cl⋯π contact in (I), the co-formers were subjected to electrostatic potential mapping through DFT-B3LYP/6-31G(*d*,*p*), as available in *CrystalExplorer17*. The analysis indicates that the Cl⋯π inter­action is weak in nature as evidenced from the white spot around the σ-hole region about the Cl1 atom in Fig. 4 ▸(*a*) as well as the faint-red spot around the centre of the π-ring centre, Fig. 4 ▸(*b*). A detailed study on the localized electrostatic charges shows that the σ-hole of Cl1 is about −0.0072 a.u. while the pyridyl π-hole is about −0.1270 a.u. indicating that the inter­action is rather dispersive in nature. This observation is in contrast with other charge complementary inter­actions as shown from the intense blue (*i.e*. electropositive) and red (*i.e*. electronegative) regions on the electrostatic surface map. For instance, the amide-N2—H2*N*⋯O2(carbon­yl) hydrogen bond has a point-to-point electrostatic charge of 0.1438 a.u. for H2*N* and −0.0622 a.u. for O2, suggestive of a strong inter­action, while benzene-C14—H14⋯O1(amide) shows complementary charges of 0.0427 and −0.0486 a.u. for H14 and O1, respectively, being indicative of a relatively weaker inter­action. Among all the identified close contacts, hydroxyl-O3—H3*O*⋯N1(pyrid­yl) is considered to be the strongest exhibiting a marked difference in the electrostatic charge of 0.2919 a.u. for H3*O* and −0.0727 a.u. for N1.

**Figure 5:**
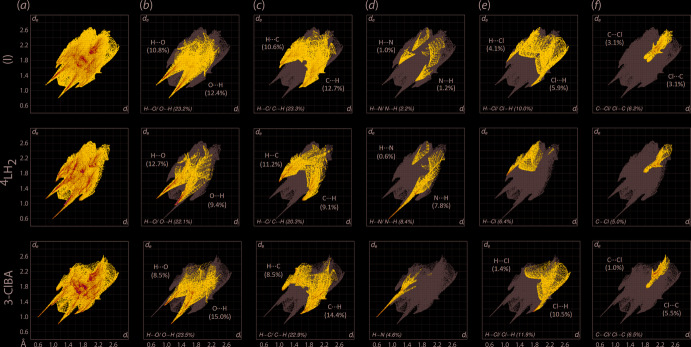
(*a*) The overall two-dimensional fingerprint plots for (I), ^4^ *L*H_2_ and 3-ClBA, and those delineated into (*b*) H⋯O/O⋯H, (*c*) H⋯C/C⋯H, (*d*) H⋯N/N⋯H, (*e*) H⋯Cl/Cl⋯H and (*f*) C⋯Cl contacts, with the percentage contributions specified within each plot.

The three-mol­ecule-aggregate of (I) as well as its individual co-formers, *i.e*. ^4^ *L*H_2_ and 3-ClBA, were subjected to fingerprint analysis for qu­anti­fication of the close contacts for each entity, Fig. 5 ▸(*a*). Overall (I) exhibits a paw-like fingerprint profile which can be delineated into H⋯H (28.5%), H⋯O/O⋯H (23.2%), H⋯C/C⋯H (23.3%), H⋯N/N⋯H (2.2%), H⋯Cl/Cl⋯H (10.0%) and C⋯Cl/C⋯Cl (6.2%), as illustrated in Fig. 5 ▸(*b*)–(*f*); others contacts amount to 6.6%, constituting contacts less than 2.0% each. Among those contacts for (I), only H⋯O/O⋯H and H⋯C/C⋯H exhibit minimum *d*_i_ + *d*_e_ contact distances tipped at *ca* 1.94 and 2.08 Å, respectively, significantly less than their respective sums of van der Waals radii of 2.61 and 2.79 Å; the remaining contacts occur at distances greater than their corresponding sums of van der Waals radii.

A similar paw-like fingerprint profile is observed for the overall fingerprint plots of the individual ^4^ *L*H_2_ and 3-ClBA mol­ecules. The key difference between these and that for (I) is the asymmetry in the distributions owing to the inter­dependency of the inter­molecular inter­actions between the two co-formers. For ^4^ *L*H_2_, the major contacts comprise H⋯H (34.5%), H⋯O/O⋯H (22.1%), H⋯C/C⋯H (20.3%), H⋯N/N⋯H (8.4%), H⋯Cl/Cl⋯H (6.4%) and C⋯Cl (5.0%). A detailed analysis on the corresponding contacts reveals that the (inter­nal)-H⋯O-(external) and (inter­nal)-H⋯C-(external) contacts are slightly more dominant over the (inter­nal)-O⋯H-(external) and (inter­nal)-C⋯H-(external) counterparts with the distribution of the contacts being 12.7 and 11.2% *versus* 9.4 and 9.1%, while the opposite is true for the (inter­nal)-H⋯N-(external) contact with a distribution of 0.6% as compared to 7.8% for (inter­nal)-N⋯H-(external). The stark difference in the dominance for H⋯N/N⋯H is likely due to the amide-H forming a hydrogen bond to O(carbon­yl) rather than to a nitro­gen acceptor. Among the major contacts, (inter­nal)-H⋯O-(external) and (inter­nal)-N⋯H-(external) display minimum *d*_i_ + *d*_e_ distances of about 1.94 and 1.60 Å, respectively, which are significantly shorter than the sums of the respective van der Waals radii as compared to the (inter­nal)-O⋯H-(external) and (inter­nal)-H⋯N-(external) counterparts of 2.24 and 3.62 Å, respectively. A similar observation is noted for (inter­nal)-H⋯C-(external) (∼2.66 Å) despite the deviation from the sum of the van der Waals radii (2.79 Å) being less significant.

As for the individual 3-ClBA mol­ecule, the major contacts in the overall fingerprint plot can be delineated into H⋯O/O⋯H (23.5%), H⋯C/C⋯H (22.9%), H⋯H (21.8%), H⋯Cl/Cl⋯H (11.9%), C⋯Cl/Cl⋯C (6.5%) and H⋯N/N⋯H (4.6%). The trend of dominance is more inclined towards (inter­nal)-*X*⋯*Y*-(external) for some close contacts (*X* = O, C and Cl; *Y* = H and C), with the distribution being 15.0, 14.4, 10.5 and 5.5% for O⋯H, C⋯H, Cl⋯H and Cl⋯C, respectively, compared to 8.5, 8.5, 1.4 and 1.0% for the corresponding H⋯O, H⋯C, H⋯Cl and C⋯Cl counterparts. In term of *d*_i_ + *d*_e_ contact distances, the key values are reciprocal to those for ^4^ *L*H_2_ owing to the inter­dependency of inter­actions as mentioned previously.

## Computational chemistry   

The calculation of the inter­action energy for all pairwise mol­ecules in (I) was performed through *CrystalExplorer17* (Turner *et al.*, 2017 ▸) following reported procedures (Tan, Jotani *et al.*, 2019 ▸) with the purpose of studying the strength of each inter­action identified from the Hirshfeld surface analysis. The results tabulated in Table 3 ▸ show that the carb­oxy­lic acid-O3—H3*O*⋯N1(pyrid­yl) hydrogen bond has the greatest inter­action energy (*E*_int_) with the value being −48.0 kJ mol^−1^, and this is followed by the dimeric amide-N2—H2*N*⋯O2(carbon­yl), benzene-C14—H14⋯O1(amide) and Cl1⋯π(N1,C1–C5) inter­actions, with a combined *E*_int_ of −38.7 kJ mol^−1^, the 16-membered {⋯OCNC_3_CH⋯} heterosynthon involving pyridyl-C1—H1⋯O1(amide) inter­actions (−24.6 kJ mol^−1^), benzene-C12—H12⋯O1(amide) and pyridyl-C4—H4⋯C11(benzene) with a combined *E*_int_ of −24.0 kJ mol^−1^, methyl­ene-C6—H6*A*⋯O3(hydrox­yl) (−15.8 kJ mol^−1^) as well as the benzene-C10⋯C10(benzene) inter­action with (−15.0 kJ mol^−1^). Inter­estingly, the strongest hydroxyl-O3—H3*O*⋯N1(pyrid­yl) inter­action in this crystal has an *E*_int_ value that is only slightly less than that of −49.4 and −52.0 kJ mol^−1^) (two independent mol­ecules) displayed by an equivalent O—H⋯N hydrogen bond complemented by a supporting pyridyl-C—H⋯O(carbon­yl) inter­action in the isomeric 2:1 co-crystal of ^4^ *L*H_2_ with 4-ClBA (Tan & Tiekink, 2020*b* ▸); the supporting C—H⋯O(carbon­yl) contact is absent in (I).

**Figure 6:**
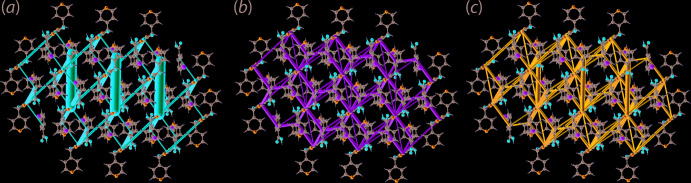
Perspective views of the energy frameworks of (I), showing the (*a*) electrostatic force, (*b*) dispersion force and (*c*) total energy. The radius of the cylinders is proportional to the relative strength of the corresponding energies, and they were adjusted to the same scale factor of 100 with a cut-off value of 8 kJ mol^−1^ within 2 × 2 × 2 unit cells.

The co-crystal system is governed by a combination of electrostatic and dispersion forces leading to a three-dimensional wire mesh-like energy framework as shown in Fig. 6 ▸. In the electrostatic energy framework, the hydroxyl-O3—H3*O*⋯N1(pyrid­yl) inter­action is the main foundation of the framework as evidenced from the thick cylindrical rods with other, relatively, thinner rods which ramify owing to various other O⋯H inter­actions, Fig. 6 ▸(*a*). The O⋯H inter­actions together with other complementary inter­actions are found to contribute to the dispersion energy framework which forms a similar topology as the electrostatic energy framework, Fig. 6 ▸(*b*). The combination of the other electrostatic and dispersion forces supersedes the strong inter­action energy from the hydroxyl-O3—H3*O*⋯N1(pyrid­yl) hydrogen bonding and leads to the overall energy framework illustrated in Fig. 6 ▸(*c*) without dominant inter­actions in a given direction. It is inter­esting to note that despite being an isomeric analogue to the ^4^ *L*H_2_·2(4-ClBA) co-crystal (Tan & Tiekink, 2020*b* ▸), (I) exhibits completely different topological frameworks as compared to the ladder-like frameworks of 4*L*H_2_·2(4-ClBA).

## Database survey   

**Figure 7:**
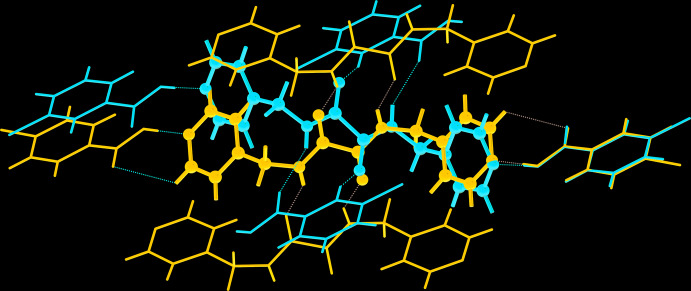
A comparison of the mol­ecular packing in (I) (red) and (II) (blue), showing the differences in the mol­ecular connectivities surrounding the central ^4^ *L*H_2_ mol­ecule.

The aforementioned analogue of (I), ^4^ *L*H_2_·2(4-ClBA) (Tan & Tiekink, 2020*b* ▸), is the most closely related, and indeed, isomeric co-crystal available for comparison; this too has been subjected to a detailed analysis of the mol­ecular packing. Co-crystals (I) and (II) are not isostructural, with the asymmetric unit of (II) comprising two half-mol­ecules of ^4^ *L*H_2_, *i.e*. ^4^ *L*H_2_-II*a* and ^4^ *L*H_2_-II*b*, as each is disposed about a centre of inversion, and two symmetry-independent mol­ecules of 4-ClBA, *i.e*. 4-ClBA-II*a* and 4-ClBA-II*b*. The common feature of the mol­ecular packing of (I) and (II) is the formation of two three-mol­ecule aggregates. The key difference in the mol­ecular packing relates to the nature of the supra­molecular tapes: in (II), the tapes are sustained by a sequence of ten-membered {⋯HNCCO}_2_ synthons, as highlighted in Fig. 7 ▸.

**Figure 8:**
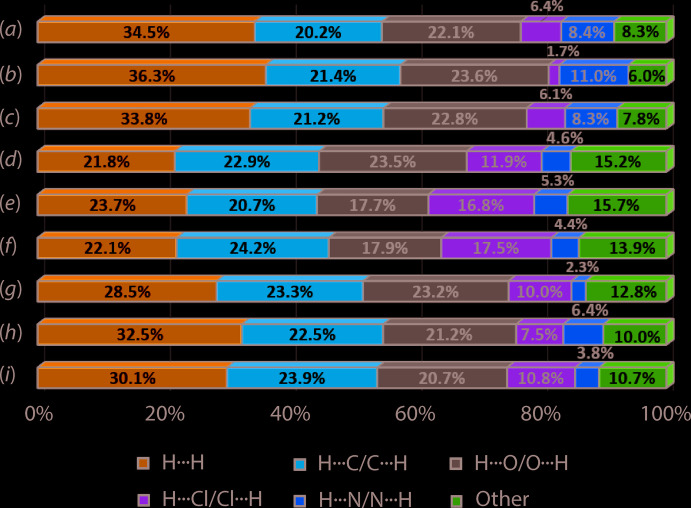
A comparison of the percentage contributions of the various contacts to the calculated Hirshfeld surfaces for (*a*) ^4^ *L*H_2_-I, (*b*) ^4^ *L*H_2_-II*a*, (*c*) ^4^ *L*H_2_-II*b*, (*d*) 3-ClBA-I, (*e*) 4-ClBA-II*a*, (*f*) 4-ClBA-II*b*, (*g*) (I), (*h*) (II*a*) and (*i*) (II*b*).

A comparison of the percentage contributions by the most prominent contacts to the respective Hirshfeld surfaces of (I) and (II), and including their individual components has been made (Jotani *et al.*, 2019 ▸). The results are summarized in Fig. 8 ▸ and suggest that to a first approximation there are no dramatic variations between the contacts made to the Hirshfeld surfaces calculated for (I) and (II). Among the noticeable differences are due to the H⋯O/O⋯H contacts which are greater for 3-ClBA, by 5.8 and 5.6%, respectively than for 4-ClBA-II*a* and II*b*. This is compensated by a reduction in the H⋯Cl/Cl⋯H contacts by 4.9 and 5.6%. One possible reason for the increase in O⋯H/H⋯O contacts in (I) *cf*. (II) relates to the participation of the carbonyl-O atom in formal hydrogen bonding to the amide-N—H group and the prominent role of the amide-O1 atom in providing points of contact between mol­ecules.

## Synthesis and crystallization   

The precursor, *N*,*N*′-bis­[(pyridin-4-yl)meth­yl]oxalamide (^4^ *L*H_2_) was prepared according to a literature procedure: M.p. 486.3–487.6 K; lit. 486–487 K (Nguyen *et al.*, 1998 ▸). 3-Chloro­benzoic acid (Merck; 3-ClBA) was of reagent grade and used as received without further purification. The co-former ^4^ *L*H_2_ (0.271 g, 0.001 mol) was mixed with 3-ClBA (0.157 g, 0.001 mol) and the mixture was then ground for 15 min in the presence of a few drops of methanol. The procedure was repeated twice. Colourless blocks were obtained through careful layering of toluene (1 ml) on an *N*,*N*-di­methyl­formamide solution (1 ml) of the ground mixture. M.p. 436.6–437.7 K. IR (cm^−1^): 3280 ν(N—H), 3070–2919 ν(C—H), 1703–1656 ν(C=O), 1524 ν(C=C), 1415 ν(C—N), 753 ν(C—Cl).

## Refinement   

Crystal data, data collection and structure refinement details are summarized in Table 4 ▸. The carbon-bound H atoms were placed in calculated positions (C—H = 0.95–0.99 Å) and were included in the refinement in the riding-model approximation, with *U*_iso_(H) set to 1.2*U*_eq_(C). The oxygen- and nitro­gen-bound H atoms were located from a difference-Fourier map and refined with O—H = 0.84±0.01 Å and N—H = 0.86±0.01 Å, respectively, and with *U*_iso_(H) set to 1.5*U*_eq_(O) or 1.2*U*_eq_(N).

## Supplementary Material

Crystal structure: contains datablock(s) I, global. DOI: 10.1107/S2056989020006568/wm5558sup1.cif

Structure factors: contains datablock(s) I. DOI: 10.1107/S2056989020006568/wm5558Isup2.hkl

CCDC reference: 2004094

Additional supporting information:  crystallographic information; 3D view; checkCIF report

## Figures and Tables

**Table 1:** Hydrogen-bond geometry (Å, °) *Cg*1 is the centroid of the (N1,C1–C5) ring.

*D*—H⋯*A*	*D*—H	H⋯*A*	*D*⋯*A*	*D*—H⋯*A*
N2—H2*N*⋯O1^i^	0.86 (2)	2.34 (3)	2.717 (2)	107 (2)
N2—H2*N*⋯O2^ii^	0.86 (2)	2.08 (2)	2.863 (2)	151 (2)
O3—H3*O*⋯N1^iii^	0.84 (2)	1.74 (2)	2.581 (2)	174 (4)
C14—H14⋯O1^iv^	0.95	2.37	3.286 (2)	161
C1—H1⋯O1^v^	0.95	2.39	3.286 (3)	157
C12—H12⋯O1^vi^	0.95	2.46	3.328 (3)	152
C6—H6*A*⋯O3	0.99	2.50	3.400 (3)	151
C13—Cl1⋯*Cg*1	1.75 (1)	3.83 (1)	5.358 (2)	145 (1)

Symmetry codes: (i) 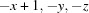; (ii) 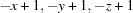; (iii) 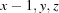; (iv) 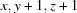; (v) 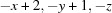; (vi) 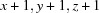.

**Table 2:** A summary of short inter­atomic contacts (Å) for (I)*^*a*^*

Contact	Distance	Symmetry operation
H2*N*⋯O2^*b*^	1.95	1 − *x*, 2 − *y*, 1 − *z*
H3*O*⋯N1^*b*^	1.60	1 − *x*, 2 − *y*, 1 − *z*
H1⋯O1	2.27	−1 + *x*, −1 + *y*, 1 + *z*
H6*A*⋯O3	2.42	−1 + *x*, −1 + *y*, 1 + *z*
H12⋯O1	2.34	1 − *x*, 1 − *y*, 1 − *z*
H14⋯O1	2.25	*x*, *y*, *z*
H4⋯C11	2.66	1 − *x*, 1 − *y*, 1 − *z*
C10⋯C10	3.28	2 − *x*, 2 − *y*, 1 − *z*

Notes: (*a*) The inter­atomic distances are calculated in *Crystal Explorer 17* (Turner *et al.*, 2017 ▸) whereby the *X*—H bond lengths are adjusted to their neutron values; (*b*) these inter­actions correspond to conventional hydrogen bonds.

**Table 3:** A summary of inter­action energies (kJ mol^−1^) calculated for (I)

Contact	*E*_ele_	*E*_pol_	*E*_dis_	*E*_rep_	*E*_tot_	Symmetry operation
N2—H2*N*⋯O2 +						
C14—H14⋯O1 +						
Cl1⋯π(N1,C1–C5)	−39.1	−6.6	−21.6	28.7	−38.7	1 − *x*, 1 − *y*, 1 − *z*
O3—H3*O*⋯N1	−102.5	−17.5	−10.2	82.2	−48.0	−1 + *x*, *y*, *z*
C1—H1⋯O1 (×2)	−22.0	−3.4	−16.1	16.9	−24.6	2 − *x*, 1 − *y*, −*z*
C6—H6*A*⋯O3	−7.7	−0.9	−18.3	11.2	−15.8	*x*, *y*, *z*
C12—H12⋯O1+						
C4—H4⋯C11	−12.9	−1.6	−31.0	21.6	−24.0	1 + *x*, 1 + *y*, 1 + *z*
C10⋯C10	−2.4	−0.4	−26.0	13.7	−15.0	2 − *x*, 2 − *y*, 1 − *z*

**Table 4:** Experimental details

Crystal data	
Chemical formula	C_7_H_5_ClO_2_·C_7_H_7_N_2_O
*M*_r_	291.71
Crystal system, space group	Triclinic, *P* 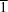
Temperature (K)	100
*a*, *b*, *c* (Å)	7.7817 (2), 9.5743 (3), 11.1516 (4)
α, β, γ (°)	113.721 (3), 90.064 (2), 112.397 (3)
*V* (Å^3^)	691.47 (4)
*Z*	2
Radiation type	Cu *K*α
μ (mm^−1^)	2.54
Crystal size (mm)	0.17 × 0.07 × 0.06

Data collection	
Diffractometer	XtaLAB Synergy, Dualflex, AtlasS2
Absorption correction	Gaussian (*CrysAlis PRO*; Rigaku OD, 2018 ▸)
*T*_min_, *T*_max_	0.604, 1.000
No. of measured, independent and observed [*I* > 2σ(*I*)] reflections	17474, 2873, 2589
*R*_int_	0.043
(sin θ/λ)_max_ (Å^−1^)	0.631

Refinement	
*R*[*F* ^2^ > 2σ(*F* ^2^)], *wR*(*F* ^2^), *S*	0.047, 0.128, 1.07
No. of reflections	2873
No. of parameters	189
No. of restraints	2
H-atom treatment	H atoms treated by a mixture of independent and constrained refinement
Δρ_max_, Δρ_min_ (e Å^−3^)	0.61, −0.47

Computer programs: *CrysAlis PRO* (Rigaku OD, 2018 ▸), *SHELXS* (Sheldrick, 2015*a* ▸), *SHELXL2017/1* (Sheldrick, 2015*b* ▸), *ORTEP-3 for Windows* (Farrugia, 2012 ▸), *DIAMOND* (Brandenburg, 2006 ▸) and *publCIF* (Westrip, 2010 ▸).

## supplementary crystallographic information

### Crystal data

**Table:**

C_7_H_5_ClO_2_·C_7_H_7_N_2_O	*Z* = 2
*M**_r_* = 291.71	*F*(000) = 302
Triclinic, *P*1	*D*_x_ = 1.401 Mg m^−^^3^
*a* = 7.7817 (2) Å	Cu *K*α radiation, λ = 1.54184 Å
*b* = 9.5743 (3) Å	Cell parameters from 7027 reflections
*c* = 11.1516 (4) Å	θ = 5.4–76.0°
α = 113.721 (3)°	µ = 2.54 mm^−^^1^
β = 90.064 (2)°	*T* = 100 K
γ = 112.397 (3)°	Rhombohedral, colourless
*V* = 691.47 (4) Å^3^	0.17 × 0.07 × 0.06 mm

### Data collection

**Table:**

XtaLAB Synergy, Dualflex, AtlasS2 diffractometer	2873 independent reflections
Radiation source: micro-focus sealed X-ray tube, PhotonJet (Cu) X-ray Source	2589 reflections with *I* > 2σ(*I*)
Mirror monochromator	*R*_int_ = 0.043
Detector resolution: 5.2558 pixels mm^-1^	θ_max_ = 76.6°, θ_min_ = 4.4°
ω scans	*h* = −9→9
Absorption correction: gaussian (CrysAlisPro; Rigaku OD, 2018)	*k* = −12→12
*T*_min_ = 0.604, *T*_max_ = 1.000	*l* = −13→13
17474 measured reflections	

### Refinement

**Table:**

Refinement on *F*^2^	Primary atom site location: dual
Least-squares matrix: full	Hydrogen site location: mixed
*R*[*F*^2^ > 2σ(*F*^2^)] = 0.047	H atoms treated by a mixture of independent and constrained refinement
*wR*(*F*^2^) = 0.128	*w* = 1/[σ^2^(*F*_o_^2^) + (0.0629*P*)^2^ + 0.363*P*] where *P* = (*F*_o_^2^ + 2*F*_c_^2^)/3
*S* = 1.07	(Δ/σ)_max_ < 0.001
2873 reflections	Δρ_max_ = 0.61 e Å^−^^3^
189 parameters	Δρ_min_ = −0.47 e Å^−^^3^
2 restraints	

### Special details

**Table:**

Geometry. All esds (except the esd in the dihedral angle between two l.s. planes) are estimated using the full covariance matrix. The cell esds are taken into account individually in the estimation of esds in distances, angles and torsion angles; correlations between esds in cell parameters are only used when they are defined by crystal symmetry. An approximate (isotropic) treatment of cell esds is used for estimating esds involving l.s. planes.

### Fractional atomic coordinates and isotropic or equivalent isotropic displacement parameters (Å^2^)

**Table:**

	*x*	*y*	*z*	*U*_iso_*/*U*_eq_	
Cl1	1.16272 (7)	1.19147 (7)	0.97323 (5)	0.04950 (19)	
O2	0.52027 (18)	0.7929 (2)	0.61549 (14)	0.0462 (4)	
O3	0.61166 (18)	0.70870 (18)	0.41896 (13)	0.0379 (3)	
H3O	0.4966 (19)	0.676 (4)	0.389 (3)	0.080 (10)*	
C8	0.6450 (2)	0.7875 (2)	0.54909 (18)	0.0323 (4)	
C9	0.8502 (2)	0.8715 (2)	0.61122 (18)	0.0314 (4)	
C10	0.9858 (3)	0.8450 (2)	0.5341 (2)	0.0337 (4)	
H10	0.949112	0.774182	0.441116	0.040*	
C11	1.1744 (3)	0.9230 (3)	0.5943 (2)	0.0383 (4)	
H11	1.266423	0.903127	0.542180	0.046*	
C12	1.2306 (3)	1.0291 (3)	0.7289 (2)	0.0385 (4)	
H12	1.360150	1.082595	0.769580	0.046*	
C13	1.0942 (3)	1.0560 (2)	0.8034 (2)	0.0358 (4)	
C14	0.9041 (2)	0.9776 (2)	0.74687 (19)	0.0341 (4)	
H14	0.812284	0.996077	0.799770	0.041*	
O1	0.62312 (17)	0.12645 (16)	−0.07710 (13)	0.0330 (3)	
N1	1.2621 (2)	0.5924 (2)	0.31316 (17)	0.0383 (4)	
N2	0.5685 (2)	0.2032 (2)	0.13447 (16)	0.0328 (3)	
H2N	0.522 (3)	0.167 (3)	0.1913 (19)	0.045 (7)*	
C1	1.1930 (3)	0.6341 (3)	0.22886 (19)	0.0388 (4)	
H1	1.278903	0.712666	0.201889	0.047*	
C2	1.0017 (3)	0.5676 (2)	0.17938 (19)	0.0365 (4)	
H2	0.958087	0.600506	0.119978	0.044*	
C3	0.8745 (3)	0.4522 (2)	0.21761 (18)	0.0339 (4)	
C4	0.9465 (3)	0.4093 (3)	0.3046 (2)	0.0444 (5)	
H4	0.863962	0.330319	0.332615	0.053*	
C5	1.1384 (3)	0.4818 (3)	0.3502 (2)	0.0459 (5)	
H5	1.185252	0.452059	0.410711	0.055*	
C6	0.6630 (3)	0.3820 (2)	0.1718 (2)	0.0381 (4)	
H6A	0.608278	0.441956	0.244426	0.046*	
H6B	0.639555	0.402602	0.094503	0.046*	
C7	0.5558 (2)	0.0917 (2)	0.01203 (17)	0.0291 (4)	

### Atomic displacement parameters (Å^2^)

**Table:**

	*U*^11^	*U*^22^	*U*^33^	*U*^12^	*U*^13^	*U*^23^
Cl1	0.0348 (3)	0.0602 (3)	0.0397 (3)	0.0143 (2)	−0.0083 (2)	0.0140 (2)
O2	0.0246 (6)	0.0723 (10)	0.0315 (7)	0.0152 (6)	0.0036 (5)	0.0177 (7)
O3	0.0292 (7)	0.0445 (8)	0.0298 (7)	0.0093 (6)	0.0013 (5)	0.0124 (6)
C8	0.0287 (9)	0.0384 (9)	0.0297 (9)	0.0112 (7)	0.0039 (7)	0.0173 (8)
C9	0.0262 (8)	0.0363 (9)	0.0333 (9)	0.0109 (7)	0.0041 (7)	0.0187 (8)
C10	0.0323 (9)	0.0355 (9)	0.0369 (10)	0.0145 (7)	0.0081 (8)	0.0189 (8)
C11	0.0286 (9)	0.0456 (11)	0.0496 (12)	0.0176 (8)	0.0127 (8)	0.0269 (10)
C12	0.0241 (8)	0.0452 (10)	0.0515 (12)	0.0113 (7)	0.0023 (8)	0.0289 (10)
C13	0.0286 (9)	0.0414 (10)	0.0371 (10)	0.0114 (7)	−0.0005 (7)	0.0199 (8)
C14	0.0259 (8)	0.0426 (10)	0.0354 (10)	0.0132 (7)	0.0043 (7)	0.0194 (8)
O1	0.0242 (6)	0.0417 (7)	0.0306 (7)	0.0080 (5)	0.0035 (5)	0.0186 (6)
N1	0.0319 (8)	0.0360 (8)	0.0355 (9)	0.0028 (6)	−0.0022 (6)	0.0155 (7)
N2	0.0246 (7)	0.0362 (8)	0.0278 (8)	0.0053 (6)	0.0000 (6)	0.0117 (6)
C1	0.0372 (10)	0.0420 (10)	0.0309 (9)	0.0058 (8)	0.0040 (8)	0.0199 (8)
C2	0.0387 (10)	0.0393 (10)	0.0290 (9)	0.0107 (8)	0.0017 (7)	0.0177 (8)
C3	0.0320 (9)	0.0301 (9)	0.0284 (9)	0.0057 (7)	−0.0022 (7)	0.0091 (7)
C4	0.0342 (10)	0.0393 (10)	0.0503 (12)	−0.0023 (8)	−0.0080 (9)	0.0273 (10)
C5	0.0380 (11)	0.0423 (11)	0.0511 (13)	0.0019 (8)	−0.0099 (9)	0.0284 (10)
C6	0.0318 (9)	0.0365 (10)	0.0366 (10)	0.0095 (8)	−0.0019 (8)	0.0117 (8)
C7	0.0169 (7)	0.0381 (9)	0.0279 (8)	0.0069 (7)	−0.0012 (6)	0.0146 (7)

### Geometric parameters (Å, º)

**Table:**

Cl1—C13	1.746 (2)	N1—C5	1.340 (3)
O2—C8	1.225 (2)	N2—H2N	0.857 (10)
O3—H3O	0.846 (10)	N2—C6	1.453 (2)
O3—C8	1.308 (2)	N2—C7	1.326 (2)
C8—C9	1.499 (2)	C1—H1	0.9500
C9—C10	1.395 (3)	C1—C2	1.385 (3)
C9—C14	1.391 (3)	C2—H2	0.9500
C10—H10	0.9500	C2—C3	1.389 (3)
C10—C11	1.386 (3)	C3—C4	1.385 (3)
C11—H11	0.9500	C3—C6	1.517 (3)
C11—C12	1.382 (3)	C4—H4	0.9500
C12—H12	0.9500	C4—C5	1.376 (3)
C12—C13	1.386 (3)	C5—H5	0.9500
C13—C14	1.386 (3)	C6—H6A	0.9900
C14—H14	0.9500	C6—H6B	0.9900
O1—C7	1.228 (2)	C7—C7^i^	1.539 (3)
N1—C1	1.340 (3)		
			
C8—O3—H3O	110 (2)	C7—N2—C6	120.72 (16)
O2—C8—O3	123.38 (17)	N1—C1—H1	118.6
O2—C8—C9	122.38 (17)	N1—C1—C2	122.84 (17)
O3—C8—C9	114.23 (15)	C2—C1—H1	118.6
C10—C9—C8	120.62 (17)	C1—C2—H2	120.4
C14—C9—C8	119.03 (16)	C1—C2—C3	119.22 (18)
C14—C9—C10	120.35 (17)	C3—C2—H2	120.4
C9—C10—H10	120.3	C2—C3—C6	121.38 (18)
C11—C10—C9	119.44 (19)	C4—C3—C2	117.76 (17)
C11—C10—H10	120.3	C4—C3—C6	120.78 (17)
C10—C11—H11	119.5	C3—C4—H4	120.2
C12—C11—C10	121.04 (18)	C5—C4—C3	119.60 (18)
C12—C11—H11	119.5	C5—C4—H4	120.2
C11—C12—H12	120.7	N1—C5—C4	122.98 (19)
C11—C12—C13	118.63 (17)	N1—C5—H5	118.5
C13—C12—H12	120.7	C4—C5—H5	118.5
C12—C13—Cl1	119.31 (15)	N2—C6—C3	112.60 (16)
C14—C13—Cl1	118.88 (15)	N2—C6—H6A	109.1
C14—C13—C12	121.81 (19)	N2—C6—H6B	109.1
C9—C14—H14	120.6	C3—C6—H6A	109.1
C13—C14—C9	118.72 (18)	C3—C6—H6B	109.1
C13—C14—H14	120.6	H6A—C6—H6B	107.8
C5—N1—C1	117.60 (17)	O1—C7—N2	124.94 (17)
C6—N2—H2N	120.9 (16)	O1—C7—C7^i^	121.2 (2)
C7—N2—H2N	118.3 (16)	N2—C7—C7^i^	113.87 (19)
			
Cl1—C13—C14—C9	179.18 (14)	N1—C1—C2—C3	−0.2 (3)
O2—C8—C9—C10	171.79 (19)	C1—N1—C5—C4	0.7 (3)
O2—C8—C9—C14	−8.8 (3)	C1—C2—C3—C4	0.1 (3)
O3—C8—C9—C10	−8.1 (2)	C1—C2—C3—C6	176.96 (19)
O3—C8—C9—C14	171.32 (17)	C2—C3—C4—C5	0.4 (3)
C8—C9—C10—C11	−179.40 (17)	C2—C3—C6—N2	138.48 (19)
C8—C9—C14—C13	−179.43 (17)	C3—C4—C5—N1	−0.8 (4)
C9—C10—C11—C12	−1.3 (3)	C4—C3—C6—N2	−44.8 (3)
C10—C9—C14—C13	0.0 (3)	C5—N1—C1—C2	−0.2 (3)
C10—C11—C12—C13	0.3 (3)	C6—N2—C7—O1	1.8 (3)
C11—C12—C13—Cl1	−179.29 (15)	C6—N2—C7—C7^i^	−177.61 (17)
C11—C12—C13—C14	1.0 (3)	C6—C3—C4—C5	−176.5 (2)
C12—C13—C14—C9	−1.1 (3)	C7—N2—C6—C3	−86.3 (2)
C14—C9—C10—C11	1.2 (3)		

Symmetry code: (i) −*x*+1, −*y*, −*z*.

### Hydrogen-bond geometry (Å, º)

*Cg*1 is the centroid of the (N1,C1–C5) ring.

**Table:**

*D*—H···*A*	*D*—H	H···*A*	*D*···*A*	*D*—H···*A*
N2—H2*N*···O1^i^	0.86 (2)	2.34 (3)	2.717 (2)	107 (2)
N2—H2*N*···O2^ii^	0.86 (2)	2.08 (2)	2.863 (2)	151 (2)
O3—H3*O*···N1^iii^	0.84 (2)	1.74 (2)	2.581 (2)	174 (4)
C14—H14···O1^iv^	0.95	2.37	3.286 (2)	161
C1—H1···O1^v^	0.95	2.39	3.286 (3)	157
C12—H12···O1^vi^	0.95	2.46	3.328 (3)	152
C6—H6*A*···O3	0.99	2.50	3.400 (3)	151
C13—Cl1···*Cg*1	1.75 (1)	3.83 (1)	5.358 (2)	145 (1)

Symmetry codes: (i) −*x*+1, −*y*, −*z*; (ii) −*x*+1, −*y*+1, −*z*+1; (iii) *x*−1, *y*, *z*; (iv) *x*, *y*+1, *z*+1; (v) −*x*+2, −*y*+1, −*z*; (vi) *x*+1, *y*+1, *z*+1.
